# SAVE: Self-Attention on Visual Embedding for Zero-Shot Generic Object Counting

**DOI:** 10.3390/jimaging11020052

**Published:** 2025-02-10

**Authors:** Ahmed Zgaren, Wassim Bouachir, Nizar Bouguila

**Affiliations:** 1 Concordia Institute for Information Systems Engineering (CIISE), Concordia University, Montréal, QC H3G 1M8, Canada; nizar.bouguila@concordia.ca; 2 Data Science Laboratory, University of Québec (TÉLUQ), Montréal, QC H2S 3L5, Canada; wassim.bouachir@teluq.ca

**Keywords:** object counting, transformers, visual attention, zero-shot, class-agnostic

## Abstract

Zero-shot counting is a subcategory of Generic Visual Object Counting, which aims to count objects from an arbitrary class in a given image. While few-shot counting relies on delivering exemplars to the model to count similar class objects, zero-shot counting automates the operation for faster processing. This paper proposes a fully automated zero-shot method outperforming both zero-shot and few-shot methods. By exploiting feature maps from a pre-trained detection-based backbone, we introduce a new Visual Embedding Module designed to generate semantic embeddings within object contextual information. These embeddings are then fed to a Self-Attention Matching Module to generate an encoded representation for the head counter. Our proposed method has outperformed recent zero-shot approaches, achieving the best Mean Absolute Error (MAE) and Root Mean Square Error (RMSE) results of 8.89 and 35.83, respectively, on the FSC147 dataset. Additionally, our method demonstrates competitive performance compared to few-shot methods, advancing the capabilities of visual object counting in various industrial applications such as tree counting, wildlife animal counting, and medical applications like blood cell counting.

## 1. Introduction

The human visual system is able to understand complex scenes. However, one of the most difficult vision tasks for humans to accomplish is counting objects in crowded scenes. Computer vision has progressively replaced manual object counting by exploiting the advantages of automation, precision, and scalability, allowing for a faster and more accurate visual object counting in various domains, such as manufacturing, retail, surveillance, and environmental monitoring.

Standard visual counting techniques were developed to count only one category of items, namely, persons [1], cells [2], and cars [3], which could often be impractical in real-world applications and are generally limited to a particular class with limited ability to generalize.

**Figure 1:**
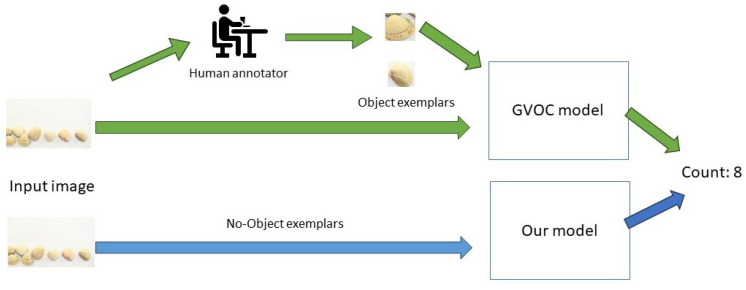
Process comparison between zero-shot and few-shot Generic Visual Object Counting (GVOC) methods.

Recently, a new approach was proposed to count objects independently of their classes, which is referred to as class-agnostic counting (CAC) [4]. We categorize CAC methods into two subcategories, zero-shot [5,6] and few-shot [7,8] methods, based on the amount of examples given to the model by the human operator (see Figure 1). On the one hand, zero-shot (or example-free) methods tend to find repetitive objects in the scene. Then, they use a similarity-based module to estimate the final count. We mainly observe two issues with zero-shot methods: finding a comprehensive, informative feature space to describe object instances in the scene, and designing a global context understanding module to match similar objects. On the other hand, few-shot methods use N∈[1,2,3] exemplars to count a target object class. The architecture of these methods generally comprises three modules: (1) an encoder module to extract features, (2) a matching module to calculate similarities between exemplar and image features, and (3) a counting module to estimate the output count. Despite important improvements, the few-shot approach presents several inherent problems. Firstly, it often requires human intervention to define the instances, which can be tedious and impractical. Furthermore, developing these models can be an intricate process, often involving several steps to achieve the desired outcome. Finally, a significant issue lies in the performance variability based on the number of instances supplied, which can lead to inconsistent results depending on the dataset used.

In this work, we propose a fully automated Generic Visual Object Counting (GVOC) method to count objects of interest of an arbitrary class, while dispensing with human intervention and external references. We present a novel pipeline that uses an end-to-end supervised training process to map an input image to the output count of the most frequent object in the scene. Specifically, we designed a counting module made up of four components: (1) a backbone, which outputs an informative feature space to describe object instances, (2) a Visual Embedding Module (VEM) to generate semantic embeddings that incorporate contextual 2D information, (3) a Self-Attention Matching Module (SAMM) to compute self-attention scores among embeddings, and (4) a Count Regression Module (CRM) to estimate the final count.

**Figure 2:**
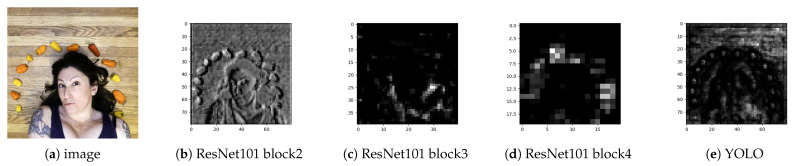
Comparison between output feature maps from a classification-based backbone, ResNet [9], and a detection-based backbone, YOLOv8 [10]. The first image from the left (**a**) is the input RGB image, extracted from the FSC147 dataset [7]. Then, (**b**–**d**) are feature maps extracted from ResNet101 [9] at different residual block levels, block2, block3, and block4, with spatial size equal to 80×80, 40×40, and 20×20, respectively. The last image (**e**) is the feature map extracted from the YOLOv8 [10] backbone at the final layer before the first detection head with spatial size 80×80.

Instead of using the classification-based backbone in existing counting models, we propose to use a detection-based backbone. We argue that a pre-trained detection-based backbone is more appropriate for extracting object instance features, while preserving spatial properties. Figure 2 shows output feature maps from two pre-trained backbones. We can observe from ResNet feature maps (see Figure 2b–d) that as we go deeper into the network, we lose spatial information of objects that are defined by the 2D properties, such as position, shape, and scale. However, features extracted from deep convolutional layers in the YOLOv8 backbone (Figure 2e) retain spatial object properties, such as relative position, shape, and scale.

Moreover, we designed a Visual Embedding Module (VEM) based on the contextual transformer architecture [11] to generate semantic embeddings incorporating 2D scene contextual information. We are inspired by the embedding layer in Natural Language Processing (NLP) when manipulating words to learn a semantic representation. The input word (converted to a token) is mapped to a unique embedding vector through the embedding layer, which is then fed to the transformer encoder. To take advantage of this property in Generic Visual Object Counting, we propose to use the spatially preserved object features to learn the image embedding by the intermediate of the contextual transformer.

After constructing the embedding of image features, we used a visual transformer-like architecture [12] to calculate the learn-long dependencies between embeddings from the input sequence. Visual Transformers have been introduced to solve many computer vision problems, such as image classification [12,13] and object detection [14]. The power behind this architecture is using the Multi-Head Self-Attention (MSA) mechanism, which outputs a new encoded representation of the input sequence that incorporates the attention scores among all the embeddings.Traditional zero-shot methods depend on precise instance localization. In contrast, our model utilizes pre-trained detection modules that learn multi-scale object-related characteristics. The combination of the detection-based backbone and the Self-Attention Matching Module offers significant advantages. The detection backbone helps the model maintain instance-level spatial information, while the Self-Attention Matching Module uses these enhanced features to identify and count repetitive objects. Finally, we used a Count Regression Module to map the final count using the encoded representation of the visual transformer.

We have demonstrated that our proposed SAVE design significantly enhances the model’s capability to identify objects without relying on reference examples. Implementing the Visual Embedding Module (VEM) improves the model’s ability to differentiate between foreground and background objects without the need for pixel-level supervision. Unlike traditional counting methods, which typically utilize density regression techniques that depend on pixel-wise supervision for object identification and counting, our model leverages spatial features derived from a detection-based backbone, leading to superior object representation. Moreover, the self-attention mechanism in the SAMM further enhances the model’s ability to distinguish between similar objects and the background in the image. We can summarize our main contributions as follows:

- We propose a novel zero-shot counting method incorporating detection information into the counting process in an end-to-end model.
- We propose a visual object embedding pipeline using a detection-based backbone to extract instance features while preserving spatial properties and a contextual transformer for generating semantic embeddings efficiently.
- We propose a simple and efficient training scheme reducing preprocessing and post-processing steps.
- Our proposed method has surpassed all other existing approaches, achieving the highest accuracy among both zero- and few-shot methods.

## 2. Related Works

### 2.1. Zero-Shot Approaches

Zero-shot counting methods aim to count the most frequent object in a given image using a fully automated mechanism. The authors in [5] proposed the first method to count objects by reference-less annotations. They suggested to identify object instances and their repetition using a self-supervised knowledge distillation and a vision transformer. Ranjan et al. [6] proposed to use Region Proposal Networks [15] to automatically identify a few exemplars from the most frequent classes in the image. For a given image, the RPN extracts region proposals and computes each proposal’s objectness and repetition scores. Then, the density predictor module computes the final density map by using the exemplar with the highest repetition score.

Zero-shot methods have the benefit of being completely automated end-to-end models that can handle human interaction. However, these methods do not always produce the best results in public datasets. The primary reason for this issue is the need to design a module that can identify the most common object in the scene. To address this problem, few-shot methods are widely used and have achieved state-of-the-art results.

### 2.2. Few-Shot Approaches

In this category, an operator is asked to annotate *N* instances of the object of interest to deliver to the model as exemplars. The model uses the exemplars as an object reference and calculates the count of that object in the image. Lu et al. [4] proposed the first class-agnostic model, the Generic Matching Network (GMN). The model has a standard architecture that sequentially combines embedding, matching, and adapting to new domains. First, a two-branch network encodes patch exemplars and input images into a feature vector and feature map. Then, a matching network learns a discriminative classifier that matches self-similar patches within the same image. Finally, the adapting stage is designed to specialize the network to new domains by implementing the residual adapter modules [16]. Ranjan et al. [7] used the correlation layer output to predict the given object’s density map. Lin et al. [17], proposed the feature correlation based on Self-Attention and Correlative-Attention modules to learn inner relations and inter-relations, respectively. Shi et al. [8] proposed BMNet+, which replaces the naive similarity metric with a bilinear similarity metric to capture flexible interactions among feature channels.

Liu et al. [18] proposed a novel transformer-based architecture whose core is a self-attention mechanism to compare visual features explicitly by using two vision transformers to encode patches and images. The resulting features are fed to a Feature Interaction Module (FIM) to fuse information from both encoders. The authors used self-supervised pre-training and supervised fine-tuning to efficiently train a model that reshapes an output embedding to a 2D map. They also applied data augmentation techniques and a test-time normalization strategy to calibrate the output density map. To address the problem of exemplars and appearance query shape while prototyping, Dukic et al. [19] proposed an iterative adaptation module. The counting process involves a two-stage architecture. Firstly, an encoder extracts features from ResNet blocks. Secondly, an Object Prototype Extraction module is employed to construct object prototypes using feature maps and bounding boxes.

Despite the results achieved by few-shot approaches, they suffer from their dependency on human-quality annotation. Model accuracy depends on three inputs: number of patches provided, annotation quality, and intra/inter-class variability.

To address these issues, we propose a new zero-shot counting model, called SAVE for Self-Attention on Visual Embedding, that exempts with the human intervention in the counting process. Our system utilizes a pre-trained detection-based backbone to generate accurate semantic spatial features for the different object instances in an image. This helps to overcome the issue of intra-class and inter-class variability that is learned by the pre-trained backbone. Additionally, our pipeline uses a self-attention mechanism to identify the frequently occurring objects and does not require a specific module or human annotations.

### 2.3. Counting by Detection

Recent methods for counting and detecting objects [20,21,22,23,24,25,26] build upon existing object detectors or segmentation techniques like [14], YOLO [10], Mask R-CNN [27], and SAM [28] to incorporate object counting capabilities. The first few-shot detection-based counter [20] was an extended transformer-based object detector [DeTR] that could identify objects specified by exemplars. Then, DAVE [21] introduced a two-stage detect-and-verify paradigm for low-shot counting and detection. In the first stage, it generates object proposals with high recall but low precision, which are then improved in a subsequent verification step. Recently, PSECO [23] proposed a three-stage approach called point segment count, which involves more sophisticated proposal generation to improve detection accuracy and also includes a verification step to enhance precision. However, this detection-based approach to counting has not yet achieved the performance levels of leading density-based counters in terms of total count estimation. To take advantages of object detection performance, we enhance the representation of objects in our model by replacing the traditional pre-trained classification backbone, like ResNet [9], by a pre-trained detection backbone.

### 2.4. Text-Guided Counting

Text-guided visual object counting has focused on leveraging multimodal learning to improve both the accuracy and flexibility of object counting. Approaches like Clip-Count [29], ZSC [30], CounTX [31], GroundingREC [32], and CountGD [33] utilize natural language descriptions to identify and count specific object instances within images, moving beyond models that are restricted to fixed classes.

These techniques often integrate vision and language models, such as transformers [34] or CLIP-based [35] architectures, to enhance the understanding and alignment of textual prompts with visual content. For example, the authors of Clip-Count use the CLIP model to generate embeddings from the input class text and the RGB image. In ZSC [30], a Variational Autoencoder (VAE) is employed to create patch prototypes from the class input text, which are then used by a trained few-shot counting model to count similar class objects within the input image.

CounTX [33] encodes a natural language description using CLIP [35] alongside a ViT [12] encoder for image feature extraction, which are then processed by a transformer encoder to facilitate feature interactions. Recently, CountGD [33] modified the GroundingDino [36] architecture to predict a density map by utilizing both the text description and visual exemplars. Additionally, the authors in REC [32] proposed counting objects based on different attributes within the same class, such as “red grape” and “green grape”. They have made modifications to GroundingDino to adapt to this requirement in the RE (Referring Expression) for the counting problem.

Accurately combining information from visual and language modalities remains a significant challenge, especially in situations that require deep cross-modal reasoning. Misalignment between textual and visual representations can hinder understanding, reducing the robustness and scalability of these methods in complex and diverse real-world applications. Our system employs a pre-trained detection-based backbone to generate precise semantic spatial features for various object instances within an image. This approach addresses the issues of intra-class and inter-class variability learned by the pre-trained backbone. Additionally, our pipeline utilizes a self-attention mechanism to identify frequently occurring objects, eliminating the need for a specific module or human annotations.

## 3. Method

### 3.1. Overview

Our method maps the input image $I\in R^{H\times W\times 3}$ to the final count C∈R using an end-to-end trainable pipeline. Our proposed architecture comprises four stages to effectively estimate the count of frequent objects in an image. The first stage involves a backbone network for extracting spatial features. In the second stage, a Visual Embedding Module (VEM) generates high-level image features and semantic embeddings through a contextual transformer. The third stage employs a Self-Attention Matching Module (SAMM) based on Multi-Head Self-Attention (MSA) to calculate the self-attention scores among all embeddings. Finally, the Count Regression Module (CRM) exploits the encoded representation to calculate the final estimated count of the frequent object.

**Figure 3:**
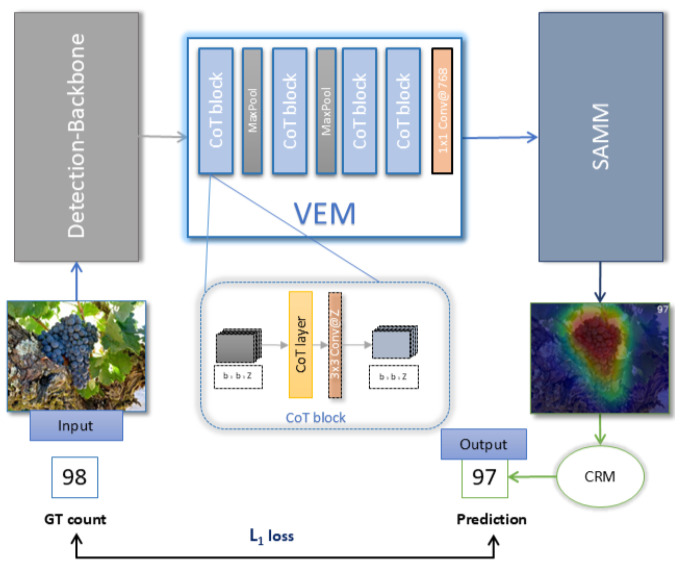
Overview of the model architecture. The input image is processed through the backbone to extract features. The VEM learns the scene context and outputs a feature map for SAMM input. The input is processed through SAMM to calculate attention scores and output the final encoded representation. The CRM estimates the final count using the encoded representation.

Figure 3 shows an overview of the architecture of the proposed model. We feed the input image *I* to the backbone, which projects it to the feature space with a dimension b×b×Z. Then, the output tensor is fed to the VEM to generate the embedding while reducing the spatial size of the feature map to fit the visual transformer fixed sequence length input, d×S. The positional embedding vector and a learnable regression embedding (learnable token) are concatenated to the generated embeddings for ViT input. The CRM uses the encoded representation to estimate the final count. This framework is trained using a simple end-to-end scheme that exempts the complex preprocessing and post-processing tasks like self-supervised pre-training and test time normalization.

### 3.2. Backbone

Object counting and detection share the task of identifying objects in an image. A successful representation model must create a semantic feature space that discriminates foreground objects from background noise, preserve their spatial properties, and maximize output map resolution. Classical backbones [9,37,38] are typically used for these tasks, but they ignore spatial properties. Modern object detection models use the Feature Pyramid Network (FPN) and Path Aggregation Network architecture (PANet) to maintain spatial properties while detecting objects at multiple scales.

In this work, we take advantages of using a pre-trained backbone on the detection. First, the input image $I\in R^{H_{o}\times W_{o}\times 3}$ is resized to H×W×3. Then, it is encoded by the backbone, which extracts spatial semantic features using *Z* channels. The backbone has three output stages for detecting small, medium, and large objects. We keep the first stage output, which has the map with the maximum spatial size b×b. Equation (1) resumes the first stage:

(1) $$M=Backbone(I_{H\times W\times 3})$$

### 3.3. Visual Object Embedding

Word embedding is an essential step in several NLP tasks. It maps each word to a vector that captures its meaning and context. These embeddings enhance the model’s ability to understand and analyze natural language data, resulting in more accurate predictions.

Inspired by word embedding in NLP, our goal is to design a module to generate image feature embeddings. The final feature map encodes each embedding using a single cell (spatial position). To achieve this, the embedding module takes the backbone output feature maps as input ($M\in R^{b\times b\times Z}$, where *b* is the width and height and *Z* is the number of channels) and maps it to the output sequence $F\in R^{s\times d}$. Here, *s* is the sequence length for visual transformer input, and *d* is the cell embedding dimension. The final sequence is the flattened version of the computed maps where each spatial cell $C\in R^{1\times d}$ is the targeted embedding vector.

**Figure 4:**
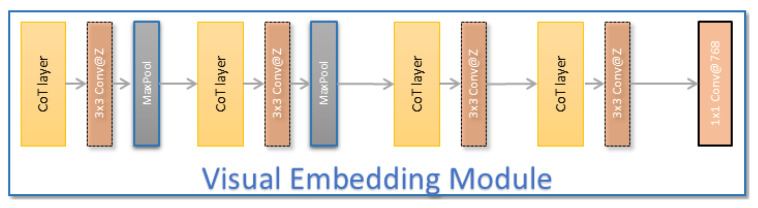
Visual Embedding Module architecture.

**Figure 5:**
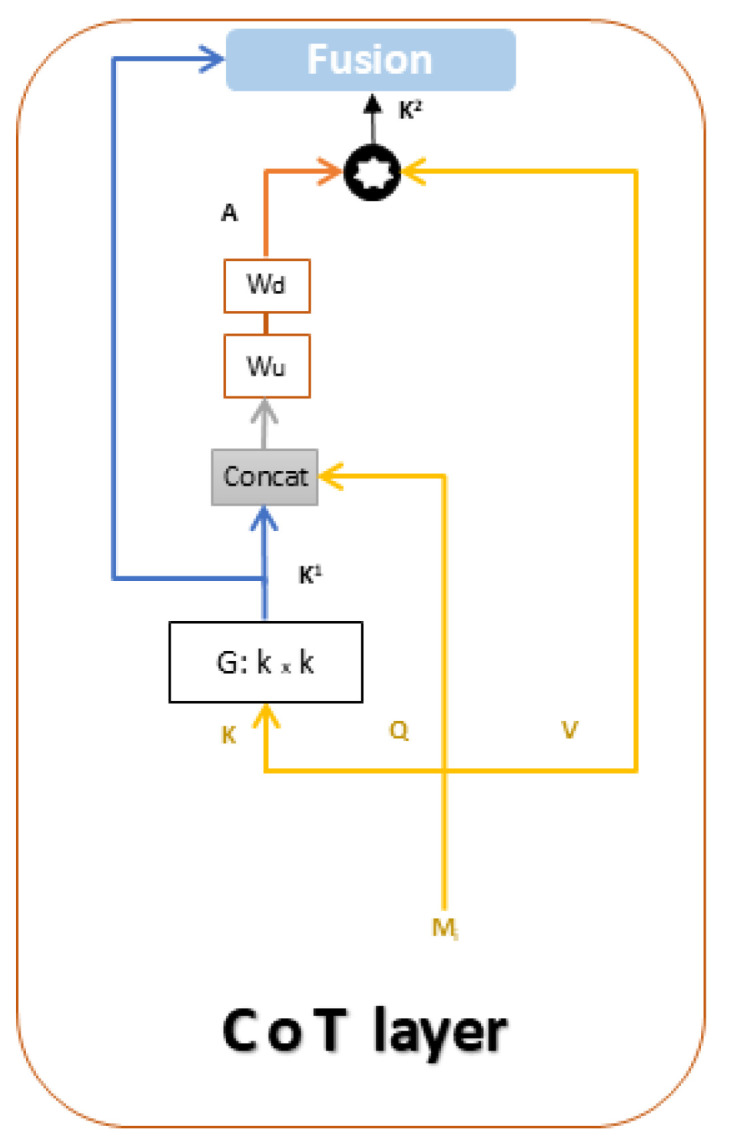
Contextual Transformer (CoT) architecture.

Our design is based on the Contextual Transformer (CoT) [11] block (see Figure 4), which has the advantage of combining self-attention learning over a 2D feature map with context mining among keys. In a CoT layer (see Figure 5), the traditional visual self-attention block [39,40] is substituted by a contextual transformer block that incorporates both contextual information mining and self-attention learning in a single architecture (see Figure 4). We define keys, queries, and values for a 2D feature map $M_{i=1...Z}$ as $K=M_{i},$$Q=M_{i},\;\mathrm{and}\;V=M_{i}.W_{v}$, respectively. In contrast to typical self-attention that encodes each key via 1×1 convolution, the CoT block first uses a $G_{k\times k}$ group convolution over all the neighboring keys within the k×k spatial grid to contextualize each key representation. We note $K^{1}$ as the static context representation of input $M_{i}$.

(2) $$K^{1}=M_{i}*G_{k\times k}$$

The attention matrix *A* is then obtained by two consecutive 1×1 convolutions ($W_{u}$ with ReLU activation function and $W_{d}$ without activation function), conditioned on the concatenation of contextualized keys $K^{1}$ and queries *Q*.

(3) $$A=\left[K^{1},Q\right]*W_{u}W_{d}$$

Furthermore, we obtain the dynamic contextual representation $K^{2}$ of input $M_{i}$ by aggregating the attention matrix *A* and values *V* as in the traditional self-attention mechanism.

(4) $$K^{2}=A*V$$

The CoT layer’s final output *Y* is the fusion of static context $K^{1}$ and dynamic context $K^{2}$ via the attention mechanism.

### 3.4. Self-Attention Matching

The matching module allows for learning the relationship between sequence embeddings through the self-attention (SA) mechanism.

Our design is based on a Visual Transformer (ViT)-like architecture [12], which follows the original NLP Transformer architecture [34] for translation tasks. The key component of the transformer encoder is the Multi-Head Self-Attention (MSA) over multi-layer architecture. Our ViT encoder inputs a sequence $X\in R^{s\times d}$. Here, *s* is the sequence length, and *d* is the embedding dimension. Then, similar to BERT’s [41] class token, we add a learnable embedding $x_{reg}\in R^{1\times d}$ at the first position of the input sequence. Before feeding the sequence to the transformer encoder, we apply position embedding to add positional information for contextual understanding. Equation (5) shows the preprocessing for the transformer input:

(5) $$H_{0}=Concat\left(x_{reg},X\right)+E_{pos}$$

The transformer encoder follows a common architecture as described in work of Vaswani et al. [34] on attention mechanisms. It consists of *N* layers, with each layer containing two blocks—the Multi-Head Self-Attention (MSA) block and the feed-forward multi-layer perceptron (MLP) block. To ensure stable training, Layer Normalization (LN) is applied before each block, and residual connections are added after each block.

(6) $$H_{l}^{\prime }=MSA\left(LN\left(H_{l-1}\right)\right)+H_{l-1},\;l=1\cdots N$$

(7) $$H_{l}=MLP\left(LN\left(H_{l}^{\prime }\right)\right)+H_{l}^{\prime },\;l=1\cdots N$$

The output of the transformer encoder $y\in R^{(s+1)\times d}$ is the result of the final layer $H_{N}$ described in Equation (8).

(8) $$y=LN(H_{N})$$

### 3.5. Count Regression

Following [42], we implemented two Count Regression Modules (CRMs). The first is the Token Regression Module (TRM), employing the LT $x_{reg}$ to forecast the final count. The second is the Sequence Regression Module (SRM), which uses the encoded sequence *y* to predict the final count.

**Token Regression Module:** The BERT’s architecture is designed to drive self-attention to effectively disseminate information among the different embeddings and the LT. As a result, the regression embedding ends up containing comprehensive semantic visual object information, which is highly valuable in various applications. We use a linear function $F:x_{reg}\to C$ to map the feature vector $x_{reg}$ to the final count *C* following Equation (9).

(9) $$C=W*x_{reg}^{T}+b$$

Here, $W\in R^{1\times d}$ is the learnable weights vector, and *b* is the learnable bias.

**Sequence Regression Module:** We ignore the LT from the output sequence $y_{0}=y-x_{reg}$ and use the Global Average Pooling (GAP) layer to reduce the sequence length to 1×d. Then, we use Equation (9) for count regression as depicted in Equation (10).

(10) $$C=W*GAP(y_{0})+b$$

### 3.6. Training

#### 3.6.1. Transfer Learning

We first fine-tune the backbone using the training set. Then, we freeze the weights while training our model, using it as a feature extraction network. However, we keep the pre-trained visual transformer encoder fully trainable to learn task-specific features. This approach of transfer learning helps the model to converge faster, even with a small dataset and standard GPU.

#### 3.6.2. Data Augmentation

Data augmentation improves model generalizability by using techniques like varying luminosity, adding noise, translation, mosaicing, and synthetic data. Mosaicing is a new technique that combines images or parts of images to generate a new image, which improves background diversity and semantic feature extraction ability among instance classes in object detection model training [10].

#### 3.6.3. Loss

In our approach, we make use of L1 loss as a metric to evaluate the dissimilarity between the predictions made by our model and the actual ground truth values. We choose the $L_{1}$ loss function because it is simple and strong for regression tasks. $L_{1}$ loss focuses on minimizing the absolute difference between predicted values and actual counts, making it less affected by outliers than $L_{2}$ loss. This is particularly important in zero-shot counting situations, where big errors in a few tough cases can greatly influence training. By penalizing differences in a straightforward way, $L_{1}$ loss supports stable and consistent learning, helping the model perform well on various and new data.

(11) $$L_{1}=\frac{1}{B}\sum_{i=1}^{B}∥C_{i}-G_{i}∥$$

Here, *B* is the batch size. $C_{i}\;\mathrm{and}\;G_{i}$ are the predicted count and the ground truth count of *i*-th image, respectively.

## 4. Experiments

First, we present an ablation analysis to examine the impact of different components of the model. We then compare it with the current state-of-the-art object counting methods, including zero-shot and few-shot counting methods. To further investigate the effectiveness of our model, we conducted a cross-dataset generalization over COCO test sets [7] and the CARPK [3] dataset.

### 4.1. Datasets

**FSC147 [7]:** We experimented on the FSC147 dataset, which contains 6135 images from 147 categories. The dataset is divided into three subsets: training, validation, and testing. Our model was trained on the train set and evaluated on the test and validation sets. Each image has annotations for a single object category, and we did not use human-annotated exemplars as our goal is to build a zero-shot counting model.

**CARPK [3]:** The CARPK dataset contains over 90,000 cars from four parking lots captured by a drone at a height of 40 m. The dataset includes bounding boxes for each car, making it great for car counting.

### 4.2. Implementation Details

We started by resizing the input image to have a height and width of 640 pixels and apply the pre-trained YOLOv8-large [10] backbone on the FSC147 training set. This generated an activation map of 80×80 pixels in size with 256 channels. We then fed this map to the VEM, which is composed of four contextual transformer blocks, resulting in a feature map of 14 by 14 pixels. This map is further projected into 768 channels by a 1×1 convolutional layer. The global self-attention block is a pre-trained visual transformer encoder [43].

To generate more training data, we applied mosaicing data augmentation on the training set, which resulted in 25,000 synthetic images. We concatenated these with the original training set to form a total of 28,546 images. We froze the backbone network parameters and trained all other model parameters for 100 epochs using the AdamW [44] optimizer with a fixed learning rate of $10^{-4}$ and weight decay of $10^{-4}$. Our model was trained on a single Nvidia RTX A6000 GPU with a batch size of 32 over a period of approximately 18 h.

### 4.3. Ablation Study

We evaluated the effectiveness of each module separately in our framework. To evaluate the role of each module, we built six different model versions. Each version was created by removing one of the studied modules and replacing it with a basic component as follows.

- $M_{0}$:We replaced the backbone and the Visual Embedding Module by a Linear Projection module.
- $M_{1}$: We replaced only the pre-trained backbone with a Linear Projection layer.
- $M_{2}$: We replaced the VEM with a ConvNet that has the same design but without the CoT blocks.
- $M_{3}$: We deleted the SAMM module and flattened the output maps. Then, we applied a GAP to fit the CRM input for the token regression module.
- $M_{TRM}$: This version comprises all the components using the TRM head.
- $M_{SRM}$: This version comprises all the components using the SRM head.

Table 1 shows the different versions of the proposed model within the valid and test results in the FSC147 dataset. During the experiments, we fixed the hyperparameters of all the modules.

**Backbone:** We observe that the use of the pre-trained backbone for detection tasks in $M_{2}\;\mathrm{and}\;M_{3}$ improves the MAE by more than 75% in both validation and test set compared to $M_{0}\;\mathrm{and}\;M_{1}$.

**VEM:** Using the VEM in $M_{1}$ enhances performance by 4% compared to $M_{0}$. Additionally, when replacing the VEM with a CNN in $M_{2}$, the MAE drops by 17%. This analysis highlights the critical role of the VEM in improving model performance to achieve the highest accuracy. We designed the VEM’s architecture to enrich feature embeddings within contextual and semantic information. The CoT layer applies the self-attention mechanism to the input feature map with a contextual window that catches neighboring information. Stacking CoT blocks assists neighboring information propagated through the feature map, which, coupled with a self-attention mechanism, enhances object features, precisely the most present objects in the scene.

**SAMM:** Applying the Self-Attention Matching Module improves the MAE by 1% and the RMSE by 11%.

**CRM:** Utilizing the encoder sequence output for the regression instead of the learned embedding token improves the MAE by 8%.

We observed that connecting the backbone to the visual embedding component significantly impacts the model’s accuracy. This confirms our assumption that creating semantic and informative image embeddings is essential.

### 4.4. Comparison with State-of-the-Art Counting Models

We present a comparison with both zero-shot counting methods and few-shot counting methods. We compare our model with the most recent generic visual counting methods on the FSC147 dataset, namely, GMN [4], MAML [45], FamNet [7], CFOCNET [46], BMNet+ [8], CounTR [18], SafeCount [47], LOCA [19], RCC [5], and RepRPN [6].

#### 4.4.1. Comparison with Zero-Shot Methods

We compare our model with methods that are designed to count with zero-shot exemplar, such as RCC [5], RepRPN [6], CounTR [18], and LOCA [19]. The results in Table 2 show that our model far outperforms zero-shot-based methods in the FSC147 dataset. We improved the MAE by 8.54 points and 49.5% in the valid set and 32.2% in the test set compared to the best recently achieved results by CounTR [18]. Moreover, our method achieved a better RMSE than LOCA with an improvement of 35% in the valid test, and 21% in the test set.

Overall, SAVE outperforms the state-of-the-art zero-shot methods, which demonstrates the effectiveness of building a self-attention counting method by integrating both a pre-trained detection backbone and a Visual Embedding Module.

#### 4.4.2. Comparison with Few-Shot Methods

To evaluate our method with respect to the state-of-the-art performance, we compare it to few-shot methods in the FSC147 dataset following the standard evaluation protocol [8,19,47]. We report, in Table 3 the best-published results from the official paper of each method using three provided exemplars. Our zero-shot proposed method outperforms all the few-shot methods in the FSC147 dataset with respect to the MAE in both the validation and test sets. We see an MAE improvement of 13% compared to the state-of-the-art method, LOCA, that uses three-exemplars as a reference to count the desired object.

Our proposed method completely automates the GVOC process and eliminates the need for human intervention. Unlike other few-shot methods that require three exemplars of an object to achieve the best accuracy, our architecture substitutes the human annotation mechanism by a self-counting mechanism. This mechanism consists of different modules that enable the counting of class-agnostic objects.

### 4.5. Cross Datasets Generalization Comparison

In this section, we evaluate the generalizability of our method on other datasets. Firstly, we present a comparison of the VAL-COCO and TEST-COCO [7], which are used by detection-based object-counting models as evaluation benchmarks. Then, we present comparison results on the object-specific counting dataset CARPK [3] to count the number of parked cars in a parking lot using the drone view.

VAL-COCO and TEST-COCO [7] are a subset of the FSC147 dataset and collected from COCO [48]. The comparison is made with both detection-based methods such as Faster R-CNN [15], Mask R-CNN [27], and RetinaNet [49], and counting-based models such as CounTR [18], FamNet [7], and LOCA [19]. Table 4 shows that our model outperforms detection-based models in the Val-COCO set and brings an improvement of 50% and 60% in MAE and RMSE, respectively. However, we observe that YOLOv8 [10] achieved a better MAE in the Test-COCO dataset and a lower RMSE. This could be explained by two reasons. The first aspect pertains to the effectiveness of multi-scale detection within the low-density scenes encountered in YOLOv8, a feature that is representative of the majority of images within the Test-COCO set. The second aspect concerns the training of YOLOv8, which was predominantly conducted and fine-tuned on the COCO large-scale dataset for object detection, resulting in its achievement of state-of-the-art performance on this dataset.

On the other hand, the bottom part of Table 4 shows that our model outperforms all the generic visual counting models in MAE metric on both Val-COCO and Test-COCO sets.

CARPK [3]: We have tested the ability of our method to generalize across different datasets using the evaluation protocol in [7]. This protocol involves training a method on the FSC147 dataset and testing it on the CARPK dataset, a car-counting dataset that contains aerial images of parking lots quite different from FSC147 images. To ensure that there is no overlap in object class between the training and test datasets, the car images are not included in the FSC147 training set. Here, GVOC methods were tested using three exemplars, and our method was tested in a fully automated scenario with zero exemplars given at test time.

We compare our method to the detection-based method, YOLOv8 [10], and state-of-the-art generic visual counting methods, FamNet [7], BMNet+ [8], CounTR [18], and LOCA [19]. All the models were firstly trained on FSC147.

Table 5 shows that our method achieved the best MAE results over cross-dataset generalization with a 9.56 mean absolute error, outperforming the recent state-of-the-art method, LOCA [19].

## 5. Qualitative Analysis

In this section, we present the qualitative results of our model on the validation and test sets of FSC147. The analysis focuses on key scenarios such as variations in object size, density, and background complexity. We also include examples of successful and failed cases to provide insight into the model’s behavior and explain the functionality of the chosen architecture. This evaluation highlights the strengths and limitations of the model while explaining its decision-making process.

**Figure 6:**
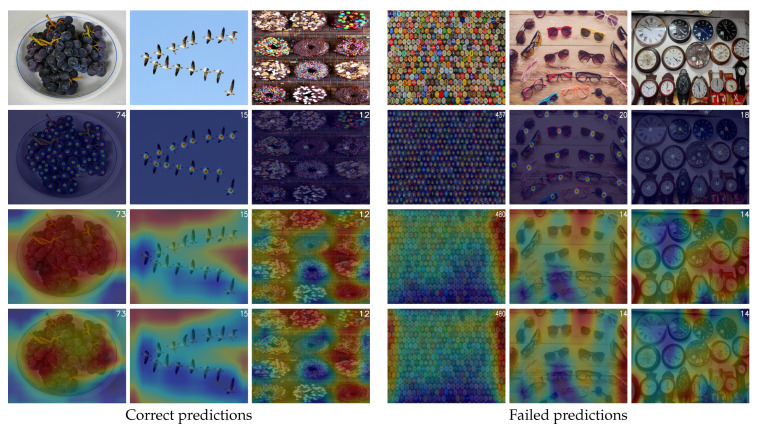
**Object Size Variation Analysis:** Qualitative results from the validation and test sets of FSC147. The first row presents the input RGB images, while the second row displays the ground truth density maps with the ground truth count indicated in the top-right corner. The third row shows the predicted count alongside the mean values of the output feature maps generated by the VEM. The fourth row illustrates the predicted count and the reshaped mean values of the output sequence produced by the SAMM. The figure is divided into two sections: the left section depicts correct predictions, and the right section highlights failed predictions. Within each section, images are further categorized into three columns corresponding to small, medium, and large object sizes, arranged from left to right. We note that the count is displayed in the top-right corner of each image.

**Object size variation:** Figure 6 illustrates examples of both correct and failed predictions, along with feature representations from the VEM and SAMM components. On one hand, the model demonstrates the ability to accurately predict the count of objects of varying sizes. Despite the absence of hard object position supervision during training, as seen in traditional methods, the third and fourth rows of the figure reveal that the model effectively distinguishes between background and object features. This highlights its capacity for spatial discrimination without explicit positional guidance.

On the other hand, the model encounters notable challenges in certain scenarios, particularly in accurately predicting object counts in highly crowded scenes with small objects. This limitation stems from the resolution of the VEM feature map, which outputs a relatively low resolution of 14×14. The reduced resolution fails to capture the fine-grained details necessary for distinguishing and encoding small objects, thereby limiting the SAMM’s ability to process these tokens effectively for counting. Furthermore, the model struggles with medium and large objects due to significant intra-class variability within certain categories, such as differences in size and shape among glass objects. This variability appears to impact the model’s ability to generalize within scenes containing diverse instances of the same class. Feature visualizations further support this observation, as they reveal that, in these cases, the model fails to adequately focus on object-specific features, instead displaying diffuse or ambiguous activations. These insights suggest opportunities for improvement, such as adopting higher-resolution feature maps or incorporating mechanisms to better handle intra-class variability.

**Figure 7:**
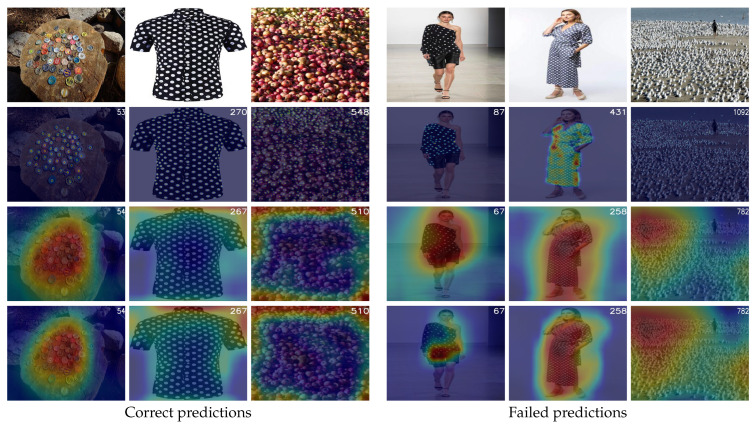
**Crowd Density Analysis:** Qualitative results from the validation and test sets of FSC147. The first row presents the input RGB images, while the second row displays the ground truth density maps with the ground truth count indicated in the top-right corner. The third row shows the predicted count alongside the mean values of the output feature maps generated by the VEM. The fourth row illustrates the predicted count and the reshaped mean values of the output sequence produced by the SAMM. The figure is divided into two sections: the left section depicts correct predictions, and the right section highlights failed predictions. Within each section, images are further categorized into three columns representing low, medium, and high crowd densities, arranged from left to right. We note that the count is displayed in the top-right corner of each image.

**Crowd Density analysis:** In this section, we analyze the model’s performance across diverse crowded scenes. Figure 7 presents qualitative results from the validation and test sets of FSC147, focusing on images with similar object sizes to provide a clearer understanding of the model’s predictions. The analysis reveals that the model accurately predicts object counts in scenes with low and medium crowd densities, even in cases with complex backgrounds and cluttered arrangements. Feature visualizations further support these observations, demonstrating that in low-density images, the model effectively focuses on regions with higher object concentrations in both VEM and SAMM components. This behavior indicates that the model identifies the most frequent object and bases its predictions on this localized representation.

However, the model struggles in highly crowded scenes with small objects and complex contexts. For example, it does not accurately count the buttons on a dress worn by a person, probably due to the overlapping nature of the objects and the complexity of the surrounding scene. These results highlight the model’s strengths in moderately crowded environments while underscoring its limitations in handling dense arrangements of small objects in intricate scenarios.

**Figure 8:**
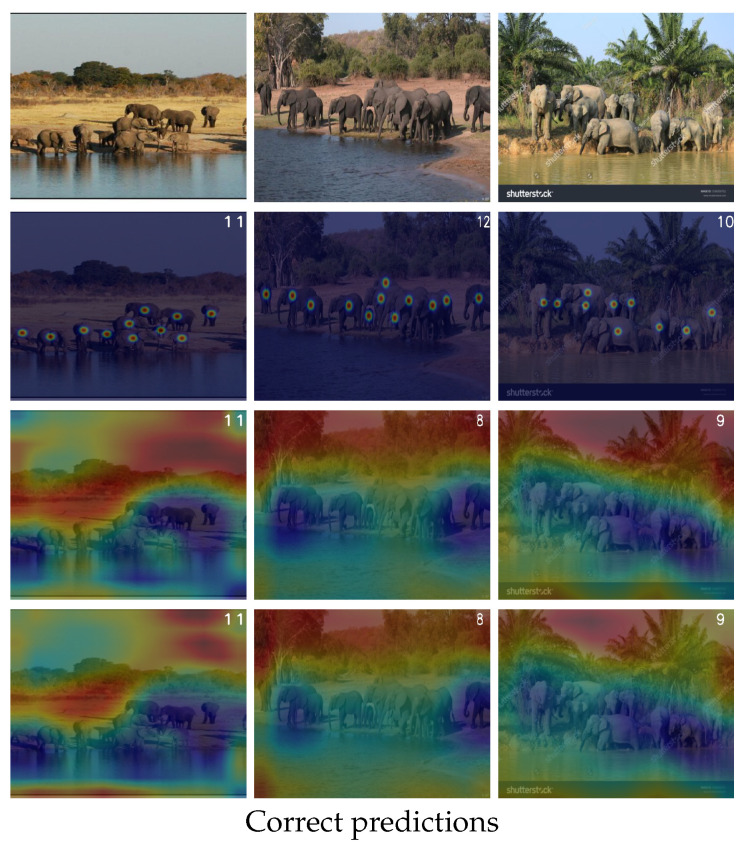
Qualitative results of a real-world scenario. Images were extracted from FSC147 validation set.

**Real-World Scenario Analysis:** To evaluate the model’s performance in real-world scenarios, we present three images from the FSC147 validation set, showcasing photographs of elephant groups in natural environments under varying challenging conditions (see Figure 8). The images exhibit diverse complexities, including variations in object size, partial occlusion, and perspective distortion. In two out of the three cases, the model accurately predicts the number of elephants. In the first image (left), despite multiple challenges such as size variation and a visually complex background, the model successfully predicts the number of elephants. Feature visualizations further demonstrate that the model effectively identifies and localizes the objects of interest, emphasizing its capability to focus on relevant regions of the scene. Similarly, in the third image (right), the model achieves a high level of accuracy, with a prediction error of less than one. This minor error is due to the complexities in the scene, such as a cluttered background with small palm trees and variations in the elephants’ orientations and sizes. However, the model encounters difficulty in the second image (center), producing a prediction error of 33%. This failure can be attributed to significant occlusion and high object clutter in the image’s central region, creating an ambiguous scenario for the model. These results highlight the model’s robustness in handling moderate real-world challenges and underline its limitations in scenarios involving severe occlusion and overlapping objects. Further refinement of the model’s ability to handle such ambiguous cases is necessary for improved performance in real-world applications.

## 6. Discussion

The integrated approach presented in this work demonstrates the capability to achieve accurate object counting without relying on reference examples or intensive pixel-level supervision. The strengths of the proposed model are evident in its ability to handle challenging scenarios such as variations in object size, background complexity, and partial occlusion, as seen in the qualitative results. The use of feature visualization provides insights into the model’s ability to localize and focus on relevant regions, even in moderately complex scenes. Additionally, the architecture’s reliance on a detection-based backbone enables efficient processing, making it well-suited for real-world applications in surveillance, environmental monitoring, inventory management, and smart agriculture. However, despite these strengths, there are notable limitations and areas for improvement:

- **Single-Class Focus:** The model is primarily designed for scenarios involving a single dominant object class, limiting its applicability in multi-class object-counting tasks. To address this, future work could explore extending the architecture to support multi-class counting by integrating a multi-label classification module or using an adaptive attention mechanism to differentiate between classes.
- **Dependency on Detection Backbone Quality:** The performance is highly reliant on the accuracy and robustness of the detection-based backbone (YOLOv8). Any deficiencies in the backbone, such as poor localization in cluttered scenes, can negatively impact the entire pipeline. To mitigate this, incorporating an ensemble of backbones or using a more advanced transformer-based backbone (e.g., Swin Transformer) could improve detection reliability and downstream counting performance.
- **Dependency on Transformer architecture:** While SAMM performs well in visual object counting, its reliance on the Vision Transformer (ViT) architecture leads to high memory and computational costs, limiting deployment on resource-constrained devices. Exploring lightweight alternatives like MobileViT [50] could enhance efficiency while maintaining accuracy. Future work could investigate hybrid models and task-specific optimizations to improve scalability, balancing performance and efficiency for broader real-world applications.
- **Challenges with Complex Scenes:** In highly cluttered or occluded environments, the model struggles to distinguish individual objects accurately. Introducing higher-resolution feature maps and leveraging multi-scale feature aggregation could help capture fine-grained details in such scenarios. Additionally, integrating occlusion-aware modules or depth estimation techniques could improve the model’s ability to separate overlapping objects.
- **Limited Contextual Understanding:** While the model excels at recognizing repetitive patterns, it lacks the ability to fully comprehend the broader scene context, leading to potential misidentifications in ambiguous situations. Future improvements could include incorporating vision-language models (e.g., CLIP) to enhance contextual reasoning by aligning visual features with semantic descriptions, enabling better handling of complex and dynamic real-world scenes.

**Real-world applications:** The proposed model is well-suited for various real-world tasks, such as monitoring animal populations in conservation efforts, managing inventory in warehouses, and analyzing crowd density in public spaces or events. For example, in forestry management, the model can assist in counting tree clusters in satellite imagery, while in urban settings, it can support traffic monitoring by accurately counting vehicles of varying sizes.

The proposed model demonstrates strong performance in specific scenarios; however, the identified limitations underscore the need for further refinement to enhance its adaptability and accuracy in complex, multi-class, and real-world environments. Future improvements, including domain-specific fine-tuning and lightweight adaptations, hold significant potential to broaden the model’s applicability and overall impact across diverse fields.

While the model offers valuable applications in areas such as wildlife monitoring and resource management, it also raises ethical concerns, including potential privacy issues in surveillance and misuse in military contexts. To mitigate these risks, responsible deployment must be ensured through strict ethical guidelines and a focus on socially beneficial applications.

## 7. Conclusions

We propose a novel zero-shot visual object counting method called SAVE: Self-Attention on Visual Embedding that fully automates the counting process of arbitrary class objects. We designed a novel architecture that incorporates a Visual Embedding Module coupled with a visual transformer for auto-identifying objects and self-count estimation in an end-to-end supervised training pipeline.

We conducted an ablation study on the model components to evaluate the importance of each component. First, the Visual Embedding Module manipulates spatial features extracted from a pre-trained detection-based backbone to generate image features embedding that incorporates 2D contextual information gathered from each embedding neighbors. Moreover, we exploited the benefits of using self-attention module on generated visual embeddings to bring out embeddings that belong to object of interest by learning embedding relationships. Finally, although our method belongs to the zero-shot category, it achieved the best state-of-the-art results over both few-shot and zero-shot counting methods for GVOC.

The proposed method has the potential to open new perspectives to improve the automation of GVOC tasks, which could be highly beneficial for a wide range of industrial applications.

## Figures and Tables

**Table 1:** Ablation study on different model components: Backbone (Bb), Linear Projection (LP), Visual Embedding Module (VEM) with the Contextual Transformer (CoT) and with a ConvNet (CNN), Self-Attention Matching Module (SAMM), Count Regression Module (CRM) using either Token Regression Module (TRM) or Sequence Regression Module (SRM).

Model	Bb	LP	VEM		SAMM	CRM		Valid		Test	
			CoT	CNN		TRM	SRM	MAE	RMSE	MAE	RMSE
$M_{0}$		×			×	×		45.79	115.53	43.09	143.57
$M_{1}$		×	×		×	×		43.7	112.98	41.27	141.99
$M_{2}$	×			×	×	×		11.44	66.52	10.61	99.42
$M_{3}$	×		×				×	9.74	45.62	10.02	102.4
$M_{TRM}$	×		×		×	×		9.72	40.62	9.05	75.11
$M_{SRM}$	×		×		×		×	8.89	35.83	8.92	80.39

**Table 2:** Evaluation on zero-shot counting in the FSC147 dataset.

Model	Valid		Test	
	MAE	RMSE	MAE	RMSE
RepRPN [6]	31.69	100.31	28.32	128.76
MAML [45]	25.54	79.44	24.9	112.68
RCC [5]	17.49	58.81	17.12	104.53
CounTR [18]	17.4	70.33	14.12	108.01
LOCA [19]	17.43	54.96	16.22	103.96
SAVE (ours)	**8.89**	**35.83**	**8.92**	**80.39**

**Table 3:** Evaluation on few-shot counting in the FSC147 dataset.

Methods	# Shots	Valid		Test	
		MAE	RMSE	RMSE	MAE
GMN [4]	3	29.66	89.81	26.52	124.57
FamNet [7]	3	23.75	69.07	22.08	99.54
CFOCNET [46]	3	21.19	61.41	22.10	112.71
BMNet+ [8]	3	15.74	58.53	14.62	91.83
SafeCount [47]	3	15.28	47.20	14.32	85.54
CounTR [18]	3	13.13	49.83	11.95	91.23
LOCA [19]	3	10.24	**32.56**	10.79	**56.97**
SAVE (ours)	0	**8.89**	35.83	**8.92**	80.39

**Table 4:** Evaluation on Val-COCO and Test-COCO sets.

Methods	Val-COCO		Test-COCO	
	MAE	RMSE	MAE	RMSE
Faster R-CNN [15]	52.79	172.46	36.20	79.59
RetinaNet [49]	63.57	174.36	52.67	85.86
Mask R-CNN [27]	52.51	172.21	35.56	80.00
YOLOv8l [10]	30.34	142.42	**5.89**	45.69
FamNet [7]	39.82	108.13	22.76	45.92
CounTR [18]	24.66	83.84	10.89	31.11
LOCA [19]	16.86	**53.22**	10.73	31.31
SAVE (ours)	**15.74**	54.77	8.15	**18.41**

**Table 5:** Evaluation on the CARPK dataset.

Method	MAE	RMSE
YOLOv8	13.54	22.15
FamNet	28.84	44.47
BMNet+	10.44	13.77
LOCA	9.97	**12.51**
SAVE (ours)	**9.56**	19.04

## Data Availability

https://github.com/AhmedZgaren/Save (accessed on 5 February 2025).
